# Supplementary material for: Discovery of Novel miRNAs in Colorectal Cancer: Potential Biological Roles and Clinical Utility
Source: Noncoding RNA. 2023 Oct 26;9(6):65. doi: 10.3390/ncrna9060065 (PMC10660700; doi:10.3390/ncrna9060065)
Supplement: Supplementary file 1 [file ncrna-09-00065-s001.zip › ncrna-2621315-supplementary-figure.pdf]

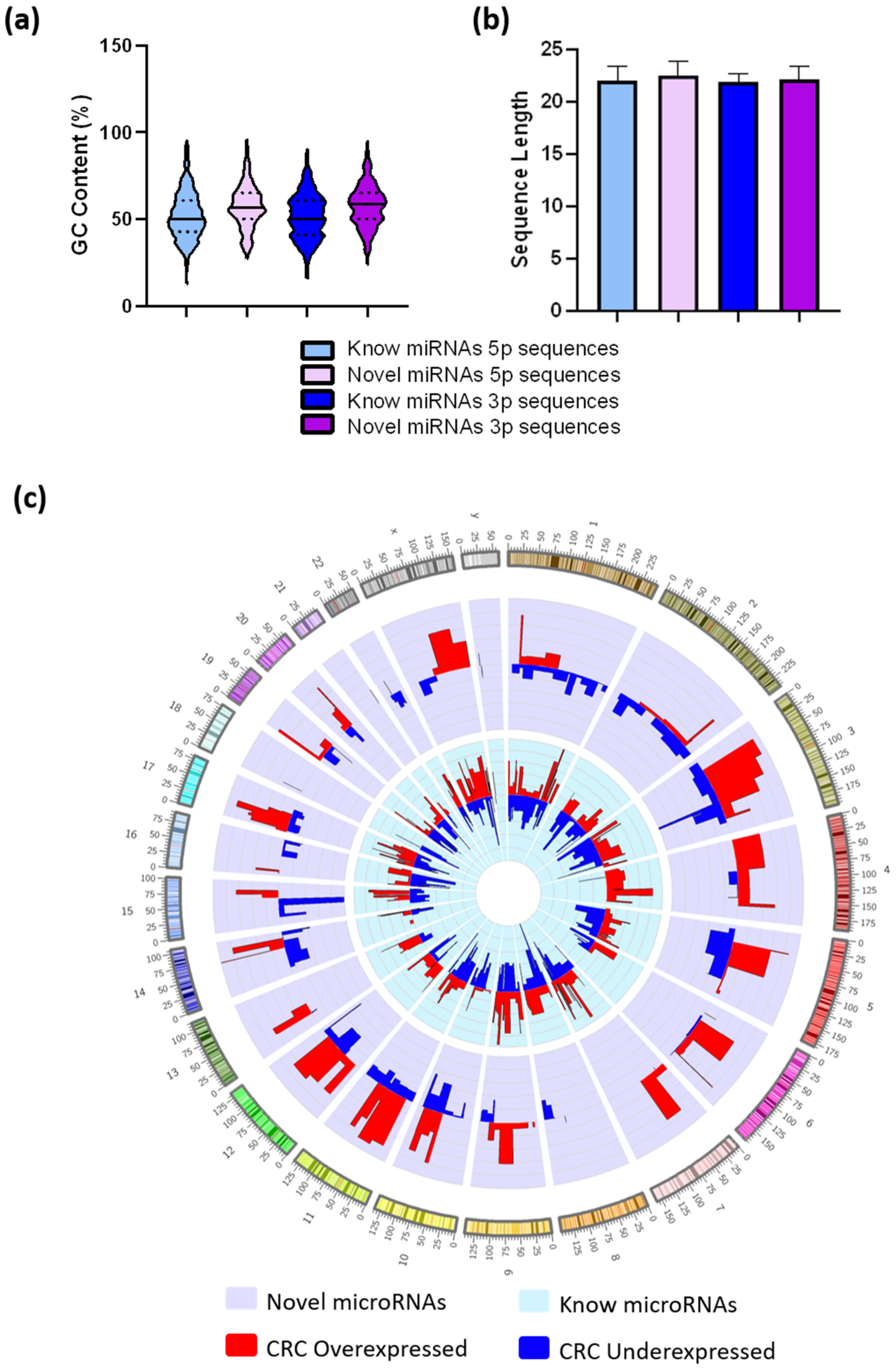

Figure S1. Structural and genomic localization characterization of novel miRNAs. (**a**) GC content of the sequences identified as novel miRNAs compared to known miRNAs present in the discovery group. (**b**) Sequence length of sequences identified as novel miRNAs compared with known miRNAs present in the discovery group. (**c**) Genomic distribution of novel and known miRNAs identified in colorectal tissues and their respective expression levels (log2 tumor/non-malignant ratio). The histogram represents the fold change values (log2) from the miRNA expression in colorectal adenocarcinoma (CRC) compared to non-malignant samples (bar widths of miRNA genomic positions were adjusted to 10 MB for illustration).The circle plot illustration of the genomic localization of the novel and known miRNAs was created using Clico (https://cgdv-upload.persistent.co.in/cgdv/).
